# Human papilloma virus genotypes associated with non-cervical HPV positive cancer development in UK and Ireland cohorts: a systematic review

**DOI:** 10.1186/s12879-025-12137-1

**Published:** 2025-12-29

**Authors:** Mary Mallon, Mohammad Albraikat, Andrew Kunzmann, Jacqueline A. James, Stephanie G. Craig

**Affiliations:** 1 Precision Medicine Centre of Excellence, Patrick G Johnston Centre for Cancer Research, Queen’s University Belfast, Belfast, BT9 7AE Northern Ireland UK; 2 School of Dentistry, Zarqa University, Zarqa, Jordan; 3 Wellcome-Wolfson Institute for Experimental Medicine, Queen’s University Belfast, Belfast, Northern Ireland UK; 4 Northern Ireland Biobank, Health Sciences Building, Queen’s University Belfast, Belfast, Northern Ireland UK; 5 Regional Molecular Diagnostics Service, Belfast Health and Social Care Trust, Belfast, Northern Ireland, UK

**Keywords:** Human papillomavirus, HPV, Cancer, Non-cervical, Genotyping

## Abstract

**Background:**

Human Papillomavirus (HPV) is an infectious agent notably associated with viral carcinogenesis of the cervix. Since 2019, the UK and Ireland have used the Gardasil-9 HPV vaccine to prevent new cases of HPV-positive cancers. This systematic review aims to assess whether the current HPV vaccination programme provides substantive protection against developing non-cervical HPV-positive cancers.

**Methods:**

Relevant studies were identified using the OVID-Medline and EMBASE databases. Screening and data extraction were conducted using the systematic review software Covidence. Risk of bias was assessed using the Hoy et al. tool, and statistical analysis was conducted using R statistical software (v 4.3.1).

**Results:**

Based on pre-defined search parameters, 4,086 papers were identified for screening. Following the title, abstract, and full-text review, data was extracted from 30 eligible studies. A total of 1,389 patients with HPV-positive cancers, with 24 unique HPV genotypes, were considered for analysis in this review. The most prevalent genotype across all patients was HPV16 (95.9%, 1,332/1,389). Genotypic diversity was notably greater in penile cancers compared to other non-cervical HPV-related cancers considered in the present study, with 21 HPV genotypes reported in this site alone compared to two in vaginal and vulvar cancers (*p* = 1.8E-3). Gardasil-9 was found to offer protection against 37.5% (9/24) of the unique HPV genotypes identified. However, if this vaccine had been available, there would have been sufficient genotype-specific protection to prevent 9 out of 10 HPV-positive cancers (97.8%, 1,359/1,389) retrospectively identified in the current study. It should be noted that significant regional differences in the heterogeneity of reported HPV genotypes (p < 1.0E-05) were illustrated, with London-based cohorts having the most HPV-positive cancers associated with unprotected HPV genotypes (4.7%, 31/658).

**Conclusion:**

This systematic review confirms HPV16 as the dominant genotype in HPV-positive cancers and highlights the genotypic diversity in non-cervical HPV-positive cancers. Moreover, while HPV vaccination using Gardasil-9 provides limited genotype-specific protection, it could have prevented nearly all HPV-positive cancers reported in eligible studies. Regional differences were minimal, but London had the highest proportion of unprotected genotypes. This suggests that while vaccination is highly effective in preventing HPV-related cancers, gaps in protection remain, particularly for less common genotypes.

**Supplementary Information:**

The online version contains supplementary material available at 10.1186/s12879-025-12137-1.

## Introduction

**Fig. 1:**
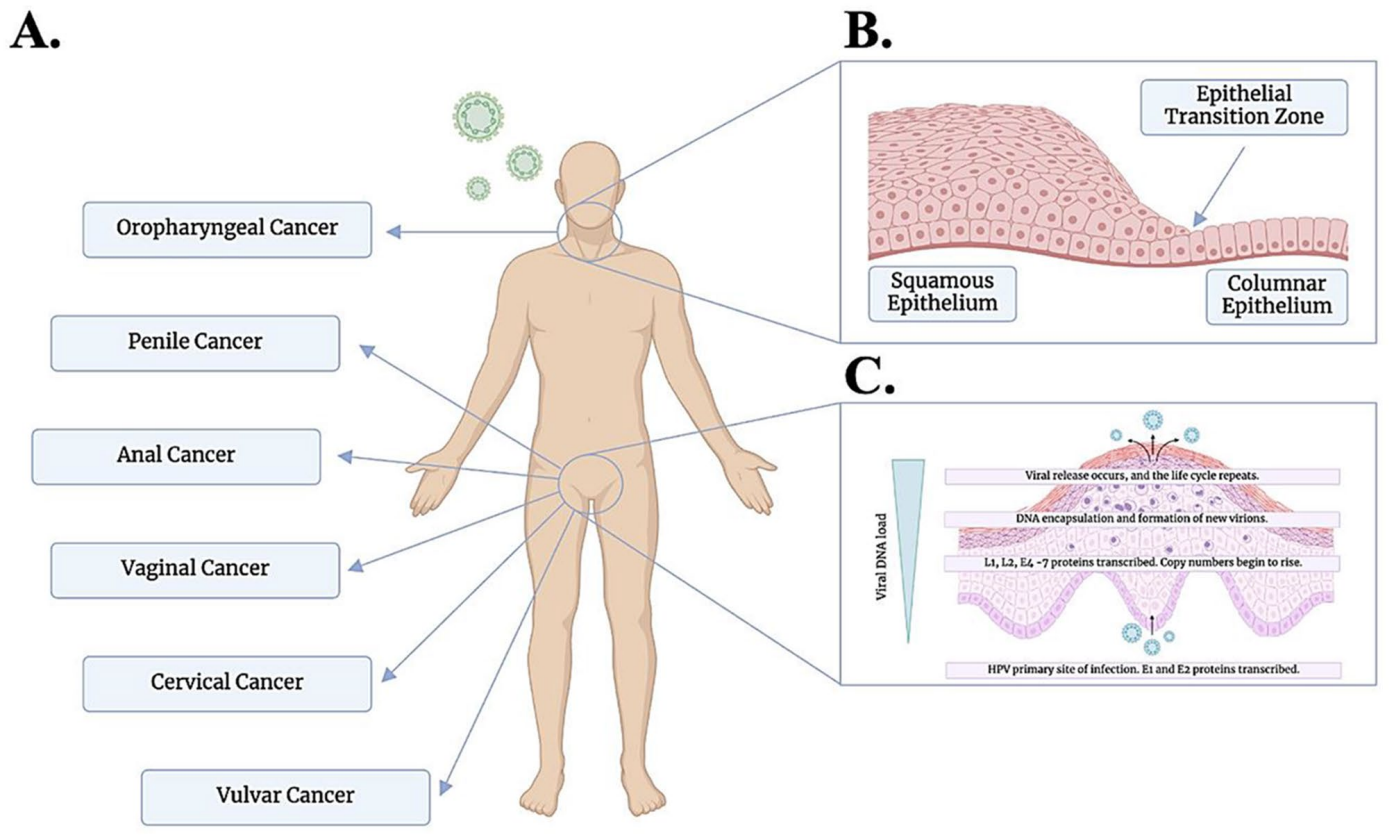
Oncogenic infective mechanism of Human Papillomavirus (HPV) at Epithelial Transition Zones (ETZ). **A**) A figure to illustrate the anatomical sites in which HPV-related cancers may develop. **B**) A figure to illustrate the histology of ETZ, wherein two opposing types of epithelia meet. These junctions are characterised by areas of epithelial remodelling and are found at a variety of anatomical sites such as the cervix, oropharynx and anogenital tract. ETZs produce micro disruptions resulting in the exposure of basal cells facilitating HPV virion entry and infection. **C**) A diagram to illustrate the mechanism of infection utilised by HPV. Upon initiation of infection within the basal cells of the epithelium, early gene products, E1 and E2, are transcribed to induce episomal replication and nuclear integration. As the infected cells differentiate and progress through the cell layers, additional viral gene products E4-7 are produced to amplify viral DNA, repress the immune response, and inactivate tumour suppressor proteins. The production of late gene products, L1 and L2, promotes virus encapsidation and the release of mature virions via cellular shedding (Figure created with https://BioRender.com)

Human Papillomavirus (HPV) is an infectious agent that has been associated with the development of multiple cancer types [1]. HPVs are small, non-enveloped, double-stranded DNA viruses that encompass over two hundred related viruses contracted primarily via sexual contact, prolonged contact with infected skin, surfaces, or even during childbirth [2]. HPV has a complex infective mechanism beginning with cellular entry with or without micro-abrasions in the epithelium [3]. HPV infection has been shown to give rise to malignancy at diverse anatomical sites, including the anus, cervix, oropharynx, penis, vagina, and vulva (Fig. 1A). A selection of these anatomical sites such as the cervix and anus are particularly susceptible to infection due to the presence of epithelial transition zones (ETZ) [4]. These are microanatomical regions where two opposing epithelia meet and are characterised by areas of epithelial remodelling. These junctions are found at a variety of anatomical sites such as the cervix, oropharynx and anogenital tract [5]. ETZs produce micro disruptions resulting in the exposure of the basal cells facilitating HPV virion entry and infection (Fig. 1B) [6]. HPV virions require direct interaction with cell surface receptors such as alpha-6-integrin to infect undifferentiated, proliferating stem cells of the epithelium found in the basal layer [7]. The gaps in the epithelium produced at ETZ can provide HPV virions access to these basal cells through a similar mechanism to micro-abrasions [8].HPV infects the cell as a circular episome of dsDNA. Early gene products, E1 and E2, are transcribed to hijack the host cell’s replication machinery for episomal replication to maintain latent infection and promote nuclear integration (Fig. 1C) [9]. During this initial phase of viral DNA replication, copy numbers are kept low (between 50 and 100 copies), and E5, E6, and E7 proteins begin to be produced [10]. These proteins are essential for promoting DNA replication and preventing apoptosis during malignant transformation. E5 prevents the activation of interferon regulatory factors in the infected cell, aiding in immune evasion [10]. The E6 protein inhibits apoptosis in infected cells via targeting the tumour suppressor protein p53 for degradation via ubiquitination, whilst the E7 protein drives the cell into the S phase of the cell cycle via inactivation of an additional tumour suppressor protein, the Rb protein [11]. As the basal cells differentiate and progress through the epithelial layers, viral DNA copy number surges via the production of E4, and late gene products, L1 and L2, are transcribed to induce virus encapsidation [12]. Mature HPV virions are then continuously shed as sloughed epithelium rather than lysed cells from the site of infection [13]. This infective mechanism reduces exposure of the viral antigens to the immune response at the site of the lesion whilst promoting loco-regional spread to nearby lymph nodes in malignant disease [10].

Infected cells eventually form koilocytes via koilocytosis, a form of cellular dysplasia that produces enlarged, darkened, and abnormally shaped nuclei with a perinuclear halo. Koilocytes are a predominant feature of active HPV infection. Persistent infection can lead to replication of the koilocytes within the upper regions of the epithelium, which phenotypically presents as a small, low-grade dysplastic lesion commonly known as a wart [10]. Most lesions will remain benign and resolve in due course. However, infection with specific HPV genotypes, of which HPV16 and HPV18 are the most prominent and well-characterised, increases the likelihood of malignant transformation [14]. HPV can be broadly classified into two groups according to cancer risk in humans: low- and high-risk [15]. According to the International Agency for Research on Cancer monograph 100B, there are four carcinogenic groups in which an HPV genotype may be placed: Group 1 (carcinogenic to humans), Group 2 A-B (probably/possibly carcinogenic to humans), Group 3 (not classifiable) and Group 4 (probably not carcinogenic to humans). Those genotypes found within group 1 (HPV16, 18, 31, 33, 35, 39, 45, 51, 52, 56, 58, 59) and group 2 (2A: HPV68, 2B: HPV26, 30, 34, 53, 66, 67, 69, 70, 73, 82) are considered ‘high-risk’, and those within group 3 (HPV40, 54, 61, 72, 84, 89) and group 4 (HPV6, 11, 42, 43, 44, 55, 81, 83) are considered ‘low-risk’ [16]. The incidence of HPV-positive cancers is increasing globally; in light of this, global vaccination programmes are now being used to protect against infection from select high-risk HPV genotypes [17]. National HPV vaccination was initially only available to young females due to the strong association with the development of cervical cancers. However, since 2019, the UK HPV vaccination programme has offered Gardasil-9 to all young people aged 9–25 [18].

Whilst the effectiveness of this vaccination strategy is well-documented in cervical cancer and has been evidenced in pre-cancerous lesions of the anus, vagina, vulva, there is currently a gap in the literature regarding its efficacy in other HPV-positive malignancies specifically [19]. Contemporary HPV vaccines protect against selected high-risk genotypes associated with cancer and low-risk genotypes related to genital warts. It is currently unknown what potential protection these vaccines could offer against developing HPV-positive cancers in non-cervical sites. This review aims to compile, present, and investigate what HPV genotypes have been reported in HPV-positive non-cervical cancers in the UK and Ireland and how they relate to HPV vaccines offered by the UK National Health Service and the Health Service Executive in Ireland. This study will address whether the current HPV vaccination programme provides substantive protection against developing HPV-positive cancer at sites other than the cervix in assumed unvaccinated individuals.

## Materials and methods

### Data sources and search strategy

A systematic review to determine all “Human Papillomavirus Genotypes Associated with Non-Cervical HPV-Positive Cancer Development in UK and Irish Cohorts” was registered with PROSPERO (CRD42024532375) on 09/04/24 and was conducted according to PRISMA guidelines (Supplementary Data S1) [20]. A specific search strategy was developed using the EMBASE and OVID-Medline databases to find studies dating from the databases’ inception until 29/02/24 based on pre-selected regional, virological, anatomical, and cancer-related keywords. Studies had to include at least one keyword or Medical Subject Heading (MeSH) term from each of the following: (i) ‘Ireland’, ‘United Kingdom’, ‘England’, ‘Scotland’, ‘Wales’, ‘Northern Ireland’; (ii) ‘Human Papillomavirus’, ‘HPV’, ‘Genotype’, ‘Subtyping’; (iii) ‘Anal’, ‘Penile’, ‘Vaginal’, ‘Vulvar’, ‘Oropharyngeal’; and (iv) ‘Cancer’, ‘Neoplasia’, and ‘Tumour/Tumor’ (Supplementary Data S2).

### Study selection

All studies retrieved by the search strategy from both databases were imported into the specialised systematic review software Covidence for study selection [21]. The retrieved studies’ titles and abstracts were screened in duplicate by two independent reviewers (MM, MA) to identify studies that potentially met the inclusion criteria. Inclusion criteria for this review required that the study cohort (1) was from UK and/or Irish patient populations; (2) included non-cervical HPV-positive cancers as previously defined; (3) made direct reference to the different HPV genotypes investigated/present and utilised any well-described or widely adopted genotyping technologies such as HPV-specific Polymerase Chain Reaction (PCR). Both cohort and individual case studies of published full texts from retrospective and prospective studies were considered for inclusion. However, eligibility was limited to studies conducted in humans only. Review articles and conference abstracts were also excluded to limit potential duplication and risk of bias in data extracted from full texts. As this systematic review focused on patients diagnosed with HPV-positive non-cervical cancers in the UK and Ireland, studies not written in the English language were excluded. Any studies which solely discussed HPV genotypes associated with cervical cancer or pre-cancerous lesions were also excluded.

Following the initial screen, any disagreement between the primary reviewers over the eligibility of studies for full-text review was resolved through discussion with at least two tertiary reviewers (SC, AK, JJ). Studies eligible for full-text review were then retrieved and independently reviewed by the two primary reviewers to determine suitability for data extraction. Any discrepancies between the primary reviewers during the full-text review were assessed by the tertiary reviewers to achieve consensus on studies suitable for data extraction.

### Data extraction

A standardised form was developed to extract data from the included studies to assess study quality and systematically synthesise evidence. Data extracted from each study included (1) the number of patients involved in each study, (2) the anatomical site studied, (3) patient cohort location, (4) genotypes reported, and 4) the genotyping method used, as well as the genotypes assessed for. If any data could not be extracted directly, missing data was requested from the corresponding author. The review team reviewed and verified the extracted data before any statistical analysis.

Study bias was assessed independently by the two primary reviewers using Covidence software [21]. The risk of bias present within studies identified for extraction was evaluated by adjusting the risk of bias tool for prevalence studies developed by Hoy et al. at an outcome level to reflect the current research question (Supplementary Data S3) [22]. A study was considered to have a high risk of bias overall if more than two of the ten elements in the tool were regarded as high risk. Of these, appropriate methodologies for HPV genotyping were considered one of the most important indicators of risk. However, within these acceptable methodologies, there can be substantial variability in assay design and applicability; therefore, a sub-analysis of these data was also conducted to determine the potential bias in HPV genotypes reported across the studies using quantitative PCR, qualitative PCR, and non-PCR-based assays. This study defines quantitative PCR as any PCR-based assay which allows for the quantification of viral DNA.

### Statistical analysis

All statistical analysis was conducted using R statistical software (v.4.3.1) [23]. All data was extracted from eligible studies at a patient level. A pooled analysis of the patient data was performed using chi-square tests to assess (1) the distribution of HPV genotypes according to the anatomical site, (2) HPV genotypes detected according to geographical location, and (3) hypothetical HPV genotype-specific protection according to vaccine regime. A p-value of < 0.05 was considered statistically significant. Bar and Pie charts were created using Microsoft Excel to support data visualisation [24].

## Results

### HPV16 was the most prevalent HPV genotype reported across the anatomical sites assessed and penile cancers presented with most diverse HPV genotypes

**Fig. 2:**
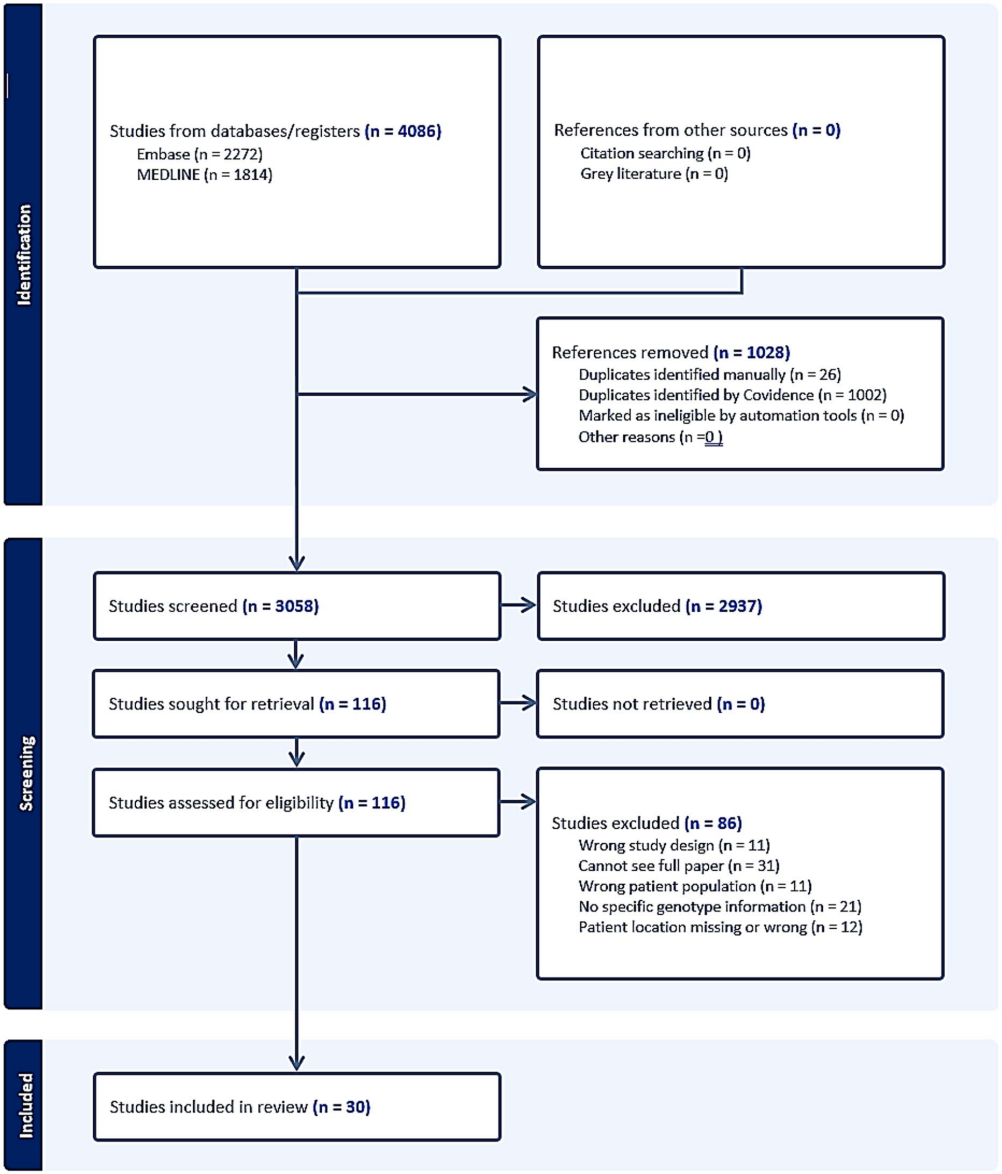
A PRISMA flow diagram. This diagram presents the number of studies identified before screening, the number of papers that met the inclusion criteria following title and abstract, and full-text screening. Figure automatically generated by Covidence software [21]

The proposed search strategy identified 4,086 records across both databases (EMBASE *n* = 2,272 and Medline *n* = 1,814) (Fig. 2). Using Covidence software, 1,028 duplicates were removed, resulting in 3,058 unique records [21]. Following title and abstract screening, 181 full texts were reviewed for eligibility and inclusion in the current study. Of these, 30 relevant articles were identified for data extraction to address the review’s aims (Supplementary Data S4) [23–52]. HPV genotyping data was extracted from these studies and summarised at a patient level for downstream statistical analysis. Due to small study numbers, HPV genotypes reported in vaginal and vulvar studies were combined (Supplementary Data S5).

Overall, 24 unique HPV genotypes were reported in 1,389 HPV-positive patients in the 30 eligible studies (Supplementary Data S6). HPV16 was reported in 95.9% (1,332/1,389) of patients as either a mono or co-infection and was found to be the most prevalent genotype reported across all sites (*p* = 7.3E-04). In 98.3% (1,379/1,389) of patients, at least one high-risk genotype was reported irrespective of site (*p* = 2.0E-02). Infection with multiple HPV genotypes was reported in a minority (3.1%, 43/1,389) of cases. However, whilst only a small number of penile cancer studies were included (*n* = 4), they were significantly more likely to report co-infection with multiple HPV genotypes than HPV-related cancers at other sites (*p* < 1.0E-04) (Table 1). 

**Table 1:**
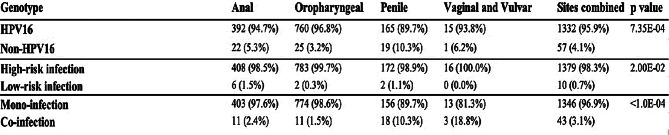
Association of reported HPV genotypes reported per patient in relation to anatomical site. All associations were assessed using the Chi-Squared test

**Fig. 3:**
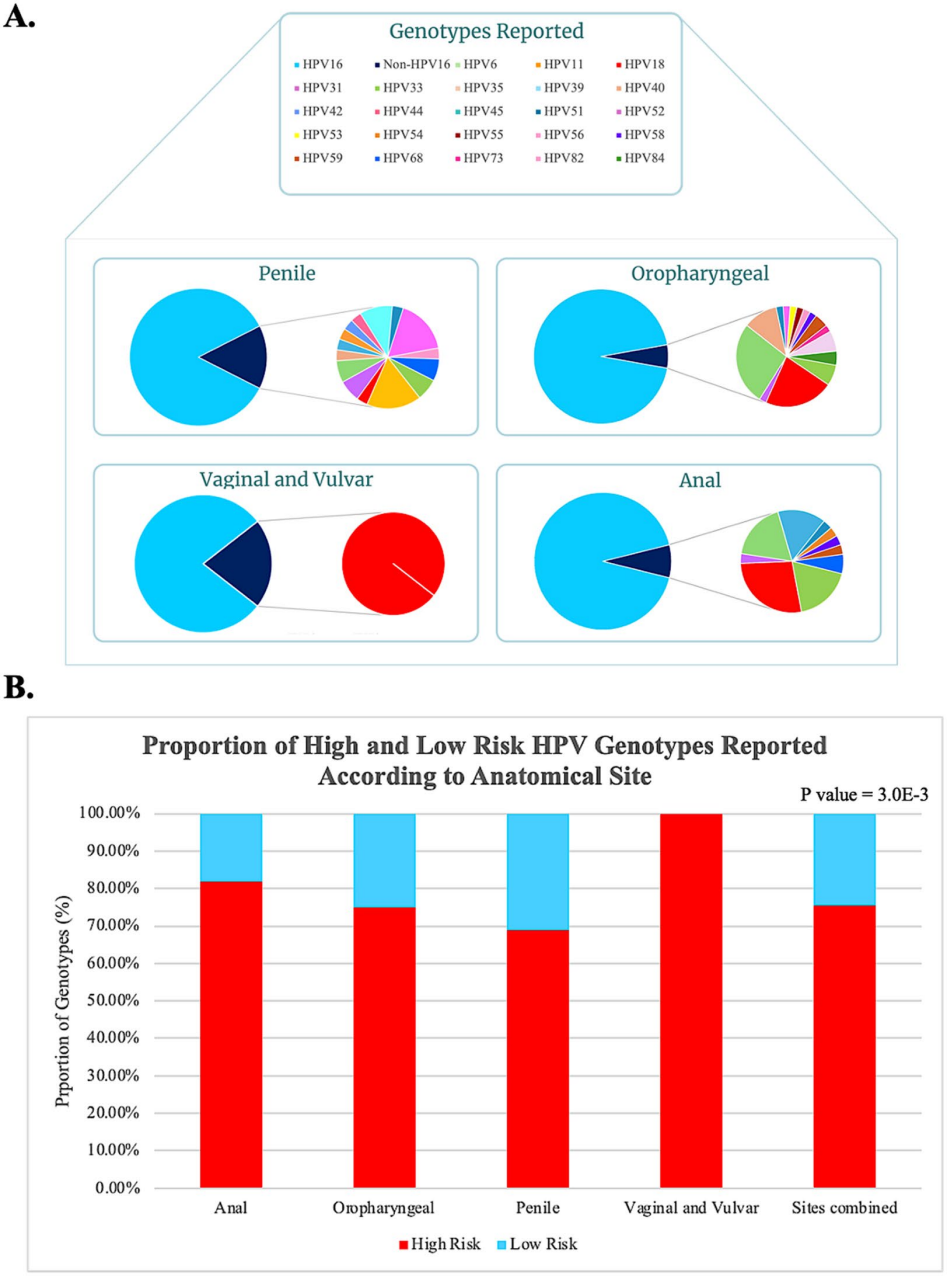
HPV genotypes reported according to anatomical site. Pie-to-Pie charts of HPV genotypes reported according to anatomical site (**A**), the initial pie chart represents the proportion of HPV16 vs. non-HPV16 genotypes reported. The expanded pie chart depicts the breakdown of non-HPV16 genotypes reported within each site. Proportional stacked bar charts and p-values generated from chi-squared tests demonstrate the proportion of low-risk and high-risk genotypes (**B**) within each site

Pie-to-Pie charts were used to visualise the proportional distribution of all HPV genotypes reported according to anatomical site (Fig. 3A). The diversity of HPV genotypes reported was notably greater in penile cancers than at other sites with 21 unique genotypes compared to 18 in oropharyngeal, 15 in anal and two in vaginal and vulvar cancers (*p* = 1.8E-3). The majority (62.5%, 15/24) of HPV genotypes reported across all sites were considered high-risk (*p* = 3.0E-3; Fig. 3B). However, the occurrence of low-risk genotypes was not found to be primarily associated with any one site (*p* = 0.9).

### HPV vaccination could provide sufficient genotype-specific protection to prevent 9 out of 10 HPV-positive cancers retrospectively identified in the current study

**Fig. 4:**
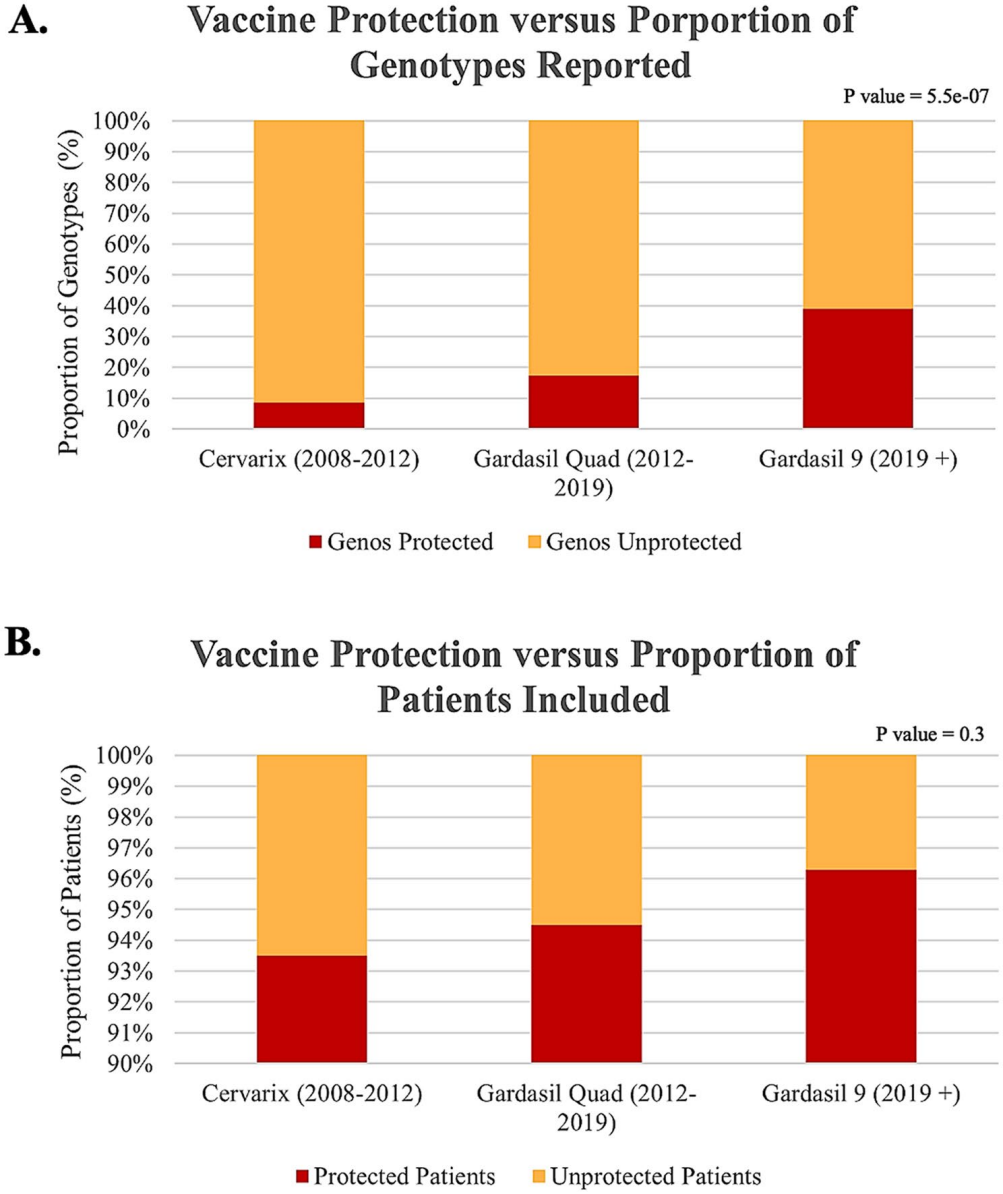
Genotype-specific protection of HPV vaccines compared to unique HPV genotypes and HPV-positive cancers reported within the UK and Ireland. Proportional stacked bar charts and p-values generated from chi-squared tests illustrate the proportion of genotype-specific protection in reported genotypes (**A**) and the hypothetical proportion of preventable HPV-related cancers (**B**) according to iterations of HPV vaccines used within the UK and Ireland

The unique HPV genotypes identified in this systematic review were compared to the various HPV vaccine formulations offered in the UK and Ireland from 2008 to date (Supplementary Data S6). A significant difference was observed in the number of HPV genotypes reported vs. genotype-specific protection offered by each iteration of the HPV vaccine (*p* = 5.5E-07; Fig. 4A). Beginning with Cervarix, an HPV vaccine introduced in 2008–2012, only 8.3% (2/24) of reported genotypes would have been protected against. Genotype-specific protection increases to 16.7% (4/24) when considering the Gardasil Quadrivalent HPV vaccine, which was introduced following Cervarix in 2012–2019. Finally, an additional increase to 37.5% (9/24) in the protection offered is seen when considering the current HPV vaccine in use within the UK and Ireland, Gardasil-9.

To determine the proportion of patients who could have hypothetically been protected from viral carcinogenesis, the HPV genotypes identified in this review were compared to each HPV vaccine. Patients with co-infections were only considered to have genotype-specific protection if all genotypes were included in the vaccine. All patients were assumed to be unvaccinated due to the included studies’ utility of retrospective cohorts from a time period prior to the implementation of the HPV vaccination programme. The proportion of protected patients exceeded 90.0% in all HPV vaccinations currently or previously offered in the UK and Ireland. No significant difference was observed in the number of HPV-related cancers reported despite a significant difference in genotype-specific protection provided by each iteration of the HPV vaccine (*p* = 0.3; Fig. 4B). Should each patient in the selected studies have received the Cervarix vaccine, 94.9% (1,319/1,389) patients could have been protected against their HPV infection. If each patient received the Gardasil Quadrivalent vaccine, patient HPV protection would increase to 95.9% (1,333/1,389). Finally, should each patient have received the most recent HPV vaccine, Gardasil-9, 97.8% (1,359/1,389) would have been offered protection against HPV-related carcinogenesis.

### There are substantial regional differences in HPV genotypes reported across the UK, with Southeast England demonstrating the most heterogeneity in HPV infections reported

**Fig. 5:**
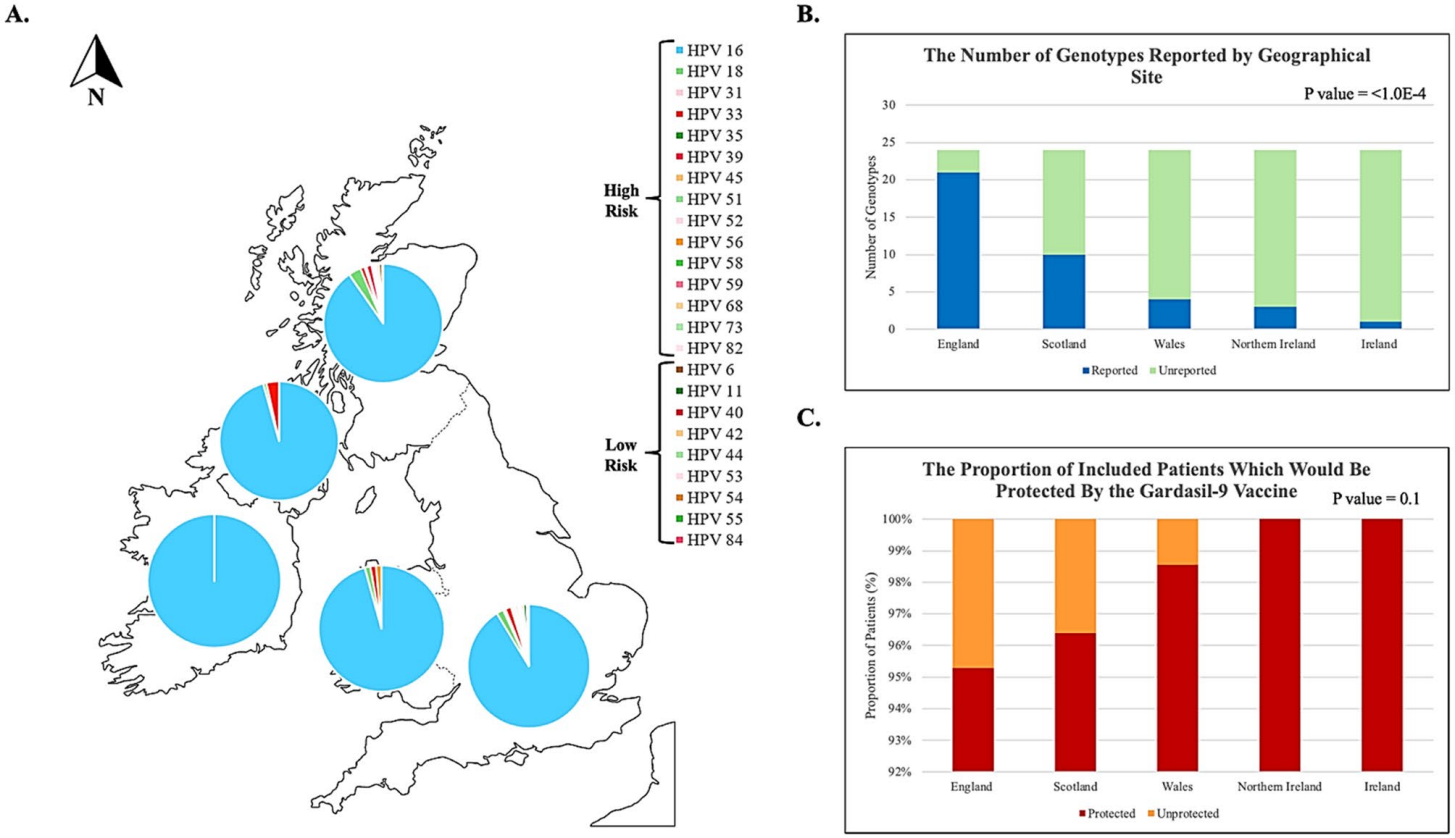
Regional heterogeneity of reported HPV genotypes within the UK and Ireland. A map illustrating the heterogeneity of unique HPV genotypes reported regionally within the UK and Ireland according to tissue source site (**A**). Proportional stacked bar charts and p-values generated from chi-squared tests illustrate the proportion of unique HPV genotypes reported (**B**) and the hypothetical proportion of unprotected HPV-related cancers (**C**) according to the tissue source site

Pie charts were used to visualise the proportional distribution of all HPV genotypes according to the geographical region where the patient samples from each study originated within the UK and Ireland (Fig. 5A). The studies with the greatest diversity of unique HPV genotypes were found to originate in England, specifically London, as this is where most English studies were situated (*p* < 1.0E-4, Fig. 5B). A wide variety of HPV genotypes were found not to be covered by the current HPV vaccine in use within the UK and Ireland, but there was no significant association of unprotected HPV infections with any geographical site (*p* = 0.1; Fig. 5C).

### The choice of assay for HPV genotyping is the most significant contributor to bias across eligible studies identified

Based on the overall aim of this systematic review, a potential risk for study bias was only found in 10% (3/30) of eligible studies identified for data extraction. However, a sub-analysis of the appropriate genotyping methodology presented in each study highlighted the potential for significant bias in the pooled analysis of the current study.

**Fig. 6:**
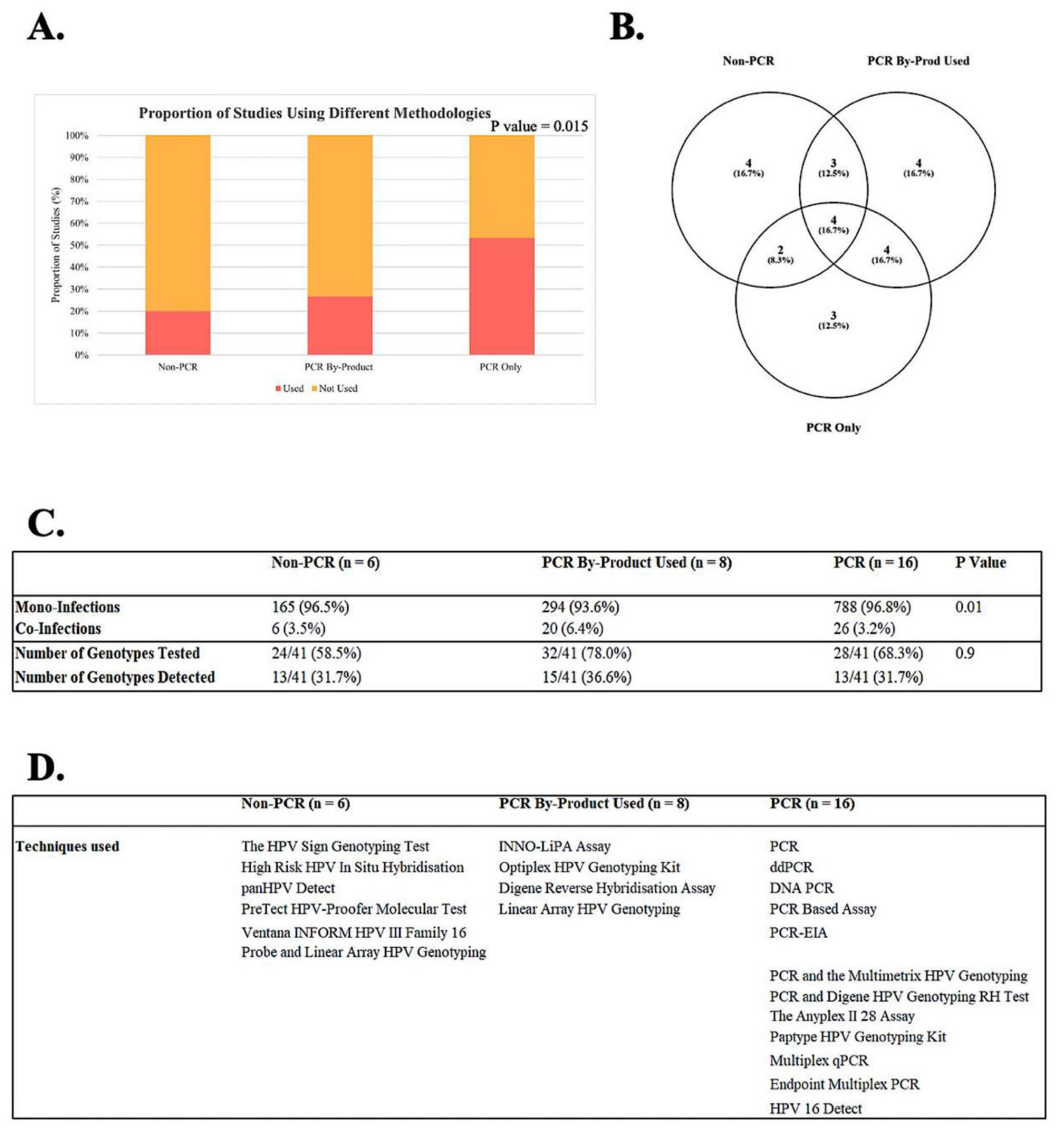
Comparison of methodology used in eligible studies to generate HPV genotyping results. A proportional stacked bar chart and p-value were generated from the chi-squared test to demonstrate the proportion of eligible studies that used either non-PCR, PCR, or PCR by-products to determine HPV genotype (**A**). Venn diagram highlighting the similarities and differences in genotype-specific testing between the various methodologies (**B**). A contingency table and p-value were generated from the chi-squared test to assess the association between the number of mono- and co-infections and the HPV genotyping method used (**C**). A table depicting the breakdown of assays used according to methodology (**D**)

In these studies, 53.3% (16/30) of eligible studies used a quantitative PCR assay to determine HPV genotype, 26.7% (8/30) used a qualitative PCR assay, and 20.0% (6/30) used a non-PCR-based method (Fig. 6A). Across all three methodologies, 41 different HPV genotypes were tested for; however, the use of these different methodologies meant that testing for only four HPV genotypes (9.8%, 4/41), HPV16, HPV18, HPV33, and HPV35, were in common across all studies eligible for data extraction (Fig. 6B). Previous studies have shown that qualitative PCR is less accurate than quantitative methods in formalin-fixed paraffin-embedded tissue [47]. In the current study, more co-infections were identified when using qualitative PCR compared to the other methods (*p* = 0.01; Fig. 6C). Moreover, the choice of the assay within each of these methodologies presents a further risk for bias, with nearly every study using a different assay (Fig. 6D).

## Discussion

This systematic review identified 30 studies that reported HPV genotypes detected in 1,389 patients in total with non-cervical HPV-positive cancers in the UK and Ireland. Following data extraction and compilation, the current study reported 24 unique HPV genotypes across all cancer sites, with site-specific differences in the genotypes. HPV-related cancers are on the rise compared to their HPV-negative counterparts at the same site [53]. Whilst numerous systematic reviews have assessed the HPV genotypes reported by site, few have noted the differences between the genotypes observed and the anatomical site [54]. The data presented in the current study highlights that whilst HPV16 remains the most frequent genotype reported in 95.9% (1,332/1,389) of patients, which is in keeping with current literature regarding the global development of HPV-positive non-cervical cancers, a broad selection of HPV genotypes is associated with viral carcinogenesis [55–57].

Based on the findings of the current study and broader literature, HPV-related cancers of both non-sex and male sex-specific origin demonstrate significant genotypic diversity and site-specific heterogeneity compared to cervical cancers. Vaccination for select HPV genotypes to prevent viral carcinogenesis was primarily offered to reduce the incidence of cervical cancer in young females [58]. Due to the enhanced knowledge surrounding HPV and cancer development in males, the UK introduced male HPV vaccination in 2019 [59]. The Gardasil-9 HPV vaccine is currently used to protect both males and females against nine different genotypes most associated with HPV-positive cancer development (HPV16, HPV18, HPV31, HPV33, HPV45, HPV52, HPV58, HPV6, and HPV11). However, uptake has been relatively poor in comparison to female vaccination rates for the same age groups. As the link between HPV and cervical cancer is so well established, global HPV vaccination in females is currently higher than in males, which, to a degree, may also be predisposing males to these HPV-positive cancers developing [60]. This has been shown in sex-specific studies of HPV-related cancer development, with men displaying increased rates globally compared to females [61]. The findings of this systematic review suggest that not only are all persons with or without a cervix at increased risk of developing non-sex-specific HPV-positive cancers due to genotypic diversity and redundancy in cancer-associated HPV genotypes, but males are particularly susceptible due to a lack of engagement with HPV vaccination due to a perceived lack of need. The results of this review should provide timely evidence to support those of the male sex to engage with vaccination where possible.

Recently, success has been noted within Scotland regarding the complete lack of cervical cancer cases developing in fully vaccinated females since the cervical cancer-targeted HPV vaccination programme began in 2008 within the UK [59]. This success is attributable to the efficacy of the numerous HPV vaccines and exemplifies the necessity for targeted vaccination and linkages between national cancer registries and vaccination records. In the UK, the Scottish Cancer Registry and the National Cancer Registration and Analysis Service currently provide commendable support in this field [62, 63]. This also illustrates the significant impact public engagement in vaccination can have as well and the time scale necessary to observe the preventative benefits of vaccination. However, these results are specific to the reduction of cervical cancers as most vaccines were designed to target HPV genotypes most frequently associated with the development of high-grade cervical lesions and cervical cancer alone. Based on the results of this systematic review, a broader diversity of HPV genotypes – beyond those included in current vaccination formulation – may play a role in non-cervical HPV-related disease. These results suggest a potential area for future vaccine development or surveillance. Despite the Gardasil-9 vaccination protecting against 37.5% (9/24) of genotypes reported here, those not currently targeted include nine high-risk genotypes (HPV35, HPV39, HPV51, HPV53, HPV56, HPV59, HPV68, HPV73, and HPV82) which can contribute to the development of malignant conditions such as those cancer types investigated here, and five low-risk genotypes (HPV40, HPV42, HPV44, HPV54, and HPV84) which can lead to the development of pre-cancerous lesions such as anogenital warts [64, 65]. Both high and low-risk HPV related disease can impact not only the daily life of a patient, but also the economic burden placed on the health service in relation to treating these conditions [66]. These results, therefore, suggest that whilst vaccination with the Gardasil-9 HPV vaccine in the UK offers substantial protection against multiple high-risk genotypes, additional untargeted genotypes may contribute to the broader spectrum of non-cervical HPV-related disease observed.

The hypothetical genotype-specific prevention of HPV-positive cancers was considered in all study patients for each iteration of the HPV vaccine offered in the UK and Ireland to determine how the genotype-specific protection of the current vaccine compares to the HPV genotypes reported in eligible studies. The analysis showed that despite the Gardasil-9 vaccine not covering every genotype reported, the hypothetical proportion of patients who would have been protected should they have received it would be 97.8% (1,359/1,389), which aligns with the current literature supporting the change from the 4- to 9-valent vaccine [67]. This poses a question as to whether a vaccine with more extensive genotype-specific protection is necessary, particularly as current vaccines offer substantial, though not complete, protection against high-grade lesions and cancers associated with the targeted HPV genotypes [67]. While most patients included in this analysis would likely be protected against from the most carcinogenic genotypes by the vaccines in use currently, clinical trial data indicates that protection against infection is lower than protection against vaccine targeted precancerous lesions or cancers [19, 68]. Whilst this high proportion of genotype-specific protection within HPV-positive cancers is advantageous, the presence of untargeted genotypes may still be of relevance. Whilst lower-risk or lower-prevalence high-risk genotypes are not of great concern in terms of cancer development, vaccinating thoroughly against current high-risk HPV genotypes may lead to a rise in the number of lower-risk or atypical high-risk HPV-positive cancers developing in the future. This highlights the need for additional research into the role of lower-risk infection in HPV-positive cancer development, as well as continued surveillance into the HPV genotypes associated with increasing HPV-positive cancer rates within the UK and Ireland [69].

Results from this systematic review demonstrate that HPV16 is the most common genotype being reported across the UK and Ireland regarding HPV-positive non-cervical cancer development. Whilst this is not a prospective HPV prevalence study, it is notable that England, specifically London-based cohorts, shows the most heterogeneity for HPV genotypes detected compared to all other areas in the UK. Furthermore, most genotypes identified in this systematic review that are not currently included in the Gardasil-9 vaccine formulation were also primarily reported in this region. Increased genotypic diversity in London could be explained by demographic variables specific to such populous cities [69]. London is one of the world’s largest business and tourism capitals, with thousands of people entering and exiting the region daily [70]. Whilst HPV infection can be spread via contact from person to person through non-sexual skin to skin contact or contact with contaminated surfaces, for example, the most documented route of transmission is through sexual intercourse [2]. The increased social traffic in populous regions may contribute to genotypic diversity and increased rates of infection within London and Southeast England, providing greater opportunity for HPV to spread from person to person [71, 72]. Therefore, the introduction of these atypical HPV subtypes, even despite some being lower-risk than others, increases the likelihood of HPV-positive cancers developing from non-targeted genotypes through positive selection by the general public.

### Limitations

This study aimed to critically appraise all HPV genotypes reported in non-cervical HPV-positive cancers within the UK and Ireland. A limitation of undertaking this study as a systematic review and meta-analysis is that across all the studies eligible for inclusion, different methodologies, specimen type, assays, and laboratory practices were used to conduct HPV genotyping. This resulted in less than 20% of genotypes being assessed across all studies due to variations in assay design. The accuracy of the results reported may also be further impacted by assay limitations for the detectability of HPV genotypes within each study, as each genotyping method carries a unique sensitivity and specificity measurement [73]. For example, many eligible studies included in this systematic review were carried out using retrospective cross-sectional cohorts. We have previously demonstrated how qualitative HPV genotyping assays are prone to false positive results in FFPE samples of increasing age and are affected by limitations in the number of genotypes that can be reliably assessed given the assay [74]. This is observed in the current study by the increased number of co-infections seen when PCR by-products are used to determine HPV status, unlike other methodologies. These assay limitations can be overcome by validating the presence of the virus in situ. However, despite the abundance of HPV genotyping assays, only one study used in situ detection of the HPV virus to validate the presence of HPV in the malignant epithelium orthogonally [74]. This is an interesting comparison to US studies, where it was common practice to confirm HPV genotypes using DNA in situ hybridisation and a series of single genotype probes [75]. In the UK, cocktail probes were permissible for clinical reporting but could not be used to determine the causative HPV genotype [76]. These factors, in addition to the preponderance of HPV16 cases reported, may explain the lack of data available within these studies regarding HPV genotypes other than HPV16. Additionally, due to most included studies utilising retrospective patient cohorts from a time period prior to the implementation of HPV vaccination programmes, all patients included in this study were assumed to be unvaccinated. Should a proportion of patients within this study have received any of the HPV-vaccines discussed, this may have reduced the frequency of certain genotypes reported/detected. Therefore, these results should be interpreted with caution. Finally, it is important to accept the potential for ‘passenger HPV infections’ which do not contribute to the viral carcinogenic process [77]. Due to this, a limitation presents in that some genotypes may have been reported which are not involved in any pathological process therefore targeting these genotypes via vaccination may not influence disease rates.

### Future research potential

This systematic review focused on well-established non-cervical HPV-related sites where the role of HPV has been clearly defined [78]. Evidence suggests that additional anatomical sites are implicated in HPV-related malignancies, such as the sinonasal tract and the oesophagus, which have not been investigated here [79, 80]. Future surveillance and prevalence studies should be conducted in emergent HPV-related cancers to ensure adequate protection against them from current vaccination strategies.

It should also be noted that whilst full integration of HPV DNA into the host cell genome is commonly seen in cervical cancers, in other HPV-positive cancers, the rate of viral integration is not well documented and was not observed in any of the eligible studies identified. This is not unexpected; the impact of HPV genetic integration into the host cell genome and its influence on outcomes is not well documented in non-cervical HPV-related cancers due to the lack of HPV screening programmes in these sites [81]. Published literature currently suggests that non-cervical HPV-positive cancers commonly exhibit a combination of integrated and extrachromosomal DNA in the form of episomes; however, this could not be assessed in relation to genotype in the studies identified [82]. This could form the basis of future research in this area to improve our knowledge of this and potentially inform clinical guidelines on the prognostication of HPV-related cancer based on their genetic episomal versus integrated state.

## Conclusion

In summary, this systematic review was designed to identify and compile all HPV genotypes reported within the UK and Ireland in relation to HPV-positive, non-cervical cancers. The main aim of this study was to evaluate the unique HPV genotypes reported in eligible studies against the genotype-specific protection provided by vaccination for HPV. This was to assess if a need exists for broader genotype-specific protection to provide additional protection if necessary. A total of 30 papers were applicable for inclusion following a targeted search of OVID-Medline and Embase databases. Of the 15 unprotected HPV genotypes reported in this systematic review that are not currently covered by the Gardasil-9 HPV vaccine utilised within the UK and Ireland, only three were considered high-risk (HPV68, HPV73 and HPV83) as they fall within Group 2 A and 2B of human carcinogens. Despite this lack of genotype-specific protection, vaccination with the Gardasil-9 vaccine could have potentially prevented viral carcinogenesis in 97.8% (1,359/1,389) of retrospectively identified HPV-positive non-cervical cancers, thereby demonstrating the relatively low incidence of unprotected HPV genotypes reported in the literature. Therefore, based on this systematic review of HPV genotypes reported in the UK and Ireland in HPV-positive non-cervical cancers, we can conclude that genotype-specific protection in the Gardasil-9 vaccine is sufficient to reduce the burden of HPV-viral carcinogenesis. However, in addition to vaccination strategies to reduce transmission and spread of this disease, surveillance of low-risk and atypical high-risk HPV genotypes in prospective studies is warranted to monitor the incidence of unprotected HPV genotypes.

## Supplementary Information

Below is the link to the electronic supplementary material.

Supplementary Material 1: PRISMA guidelines checklist for this systematic review.

Supplementary Material 2: The targeted search strategy used to interrogate EMBASE and OVID-Medline databases.

Supplementary Material 3: Risk of bias assessment questions used in the current study.

Supplementary Material 4: Table containing data extracted from all eligible studies included in this systematic review.

Supplementary Material 5: Table summarising HPV genotype data extracted from Supplementary Data S4 at a patient level for each anatomical site.

Supplementary Material 6: Table summarising genotype-specific protection of HPV vaccinations offered in relation to reported HPV genotype data extracted from retrospective HPV-positive cancers in the current study.

## Data Availability

Not applicable.
