# Supplementary material for: Human papilloma virus genotypes associated with non-cervical HPV positive cancer development in UK and Ireland cohorts: a systematic review
Source: BMC Infect Dis. 2025 Dec 29;25:1758. doi: 10.1186/s12879-025-12137-1 (PMC12752435; doi:10.1186/s12879-025-12137-1)
Supplement: Supplementary file 2 — Supplementary Material 2: The targeted search strategy used to interrogate EMBASE and OVID-Medline databases. [file 12879_2025_12137_MOESM2_ESM.docx]

**Supplementary 2 (S2): Unique search strategy word document**

| **Medline and Ovid** |
| --- |
| **HPV terms** |
| 1. papillomaviridae .mp |
| 2. papilloma .mp |
| 3. HPV .mp |
| 4. papillomavirus .mp |
| **Cancer terms** |
| 5. carcinoma .mp |
| 6. neoplasm |
| 7. cancer.mp |
| 8. neoplasms |
| 9. carcinoma |
| 10. neoplasia .mp. |
| 11. malignancy .mp |
| 12. malignant .mp |
| 13. “carcinoma in situ” |
| 14. CIN |
| 15. squamous |
| **Regional terms** |
| 16. England |
| 17. Scotland |
| 18. Wales |
| 19. Northern Ireland |
| 20. United Kingdom |
| 21. UK |
| 22. Dublin |
| 23. Mayo |
| 24. Galway |
| 25. London |
| 26. Liverpool |
| 27. Plymouth |

| 28. Bristol |
| --- |
| 29. Brighton |
| 30. Cambridge |
| 31. Oxford |
| 32. Manchester |
| 33. Edinburgh |
| 34. Glasgow |
| 35. Stirling |
| **Genotype terms** |
| 36. testing |
| 37. assay |
| 38. subtyping |
| 39. genotyping |
| 40. subtype |
| 41. typing |
| 42. PCR |
| 43. “polymerase chain reaction” |
